# Acquiring Chondrocyte Phenotype from Human Mesenchymal Stem Cells under Inflammatory Conditions

**DOI:** 10.3390/ijms151121270

**Published:** 2014-11-17

**Authors:** Masahiro Kondo, Kunihiro Yamaoka, Yoshiya Tanaka

**Affiliations:** 1 The First Department of Internal Medicine, University of Occupational and Environmental Health, 1-1 Iseigaoka, Yahatanishi-ku, Kitakyushu, Fukuoka 807-8555, Japan; E-Mails: Kondo.Masahiro@mc.mt-pharma.co.jp (M.K.); yamaoka@med.uoeh-u.ac.jp (K.Y.); 2 Pharmacology Research Laboratories I, Research Division, Mitsubishi Tanabe Pharma Corporation, 1000 Kamoshida-cho, Aoba-ku, Yokohama, Kanagawa 227-0033, Japan; 3 Division of Rheumatology, Department of Internal Medicine, School of Medicine, Keio University, 35 Shinanomachi, Shinjuku-ku, Tokyo 160-8582, Japan

**Keywords:** cartilage, chondrogenesis, mesenchymal stem cells, inflammation

## Abstract

An inflammatory milieu breaks down the cartilage matrix and induces chondrocyte apoptosis, resulting in cartilage destruction in patients with cartilage degenerative diseases, such as rheumatoid arthritis or osteoarthritis. Because of the limited regenerative ability of chondrocytes, defects in cartilage are irreversible and difficult to repair. Mesenchymal stem cells (MSCs) are expected to be a new tool for cartilage repair because they are present in the cartilage and are able to differentiate into multiple lineages of cells, including chondrocytes. Although clinical trials using MSCs for patients with cartilage defects have already begun, its efficacy and repair mechanisms remain unknown. A PubMed search conducted in October 2014 using the following medical subject headings (MeSH) terms: mesenchymal stromal cells, chondrogenesis, and cytokines resulted in 204 articles. The titles and abstracts were screened and nine articles relevant to “inflammatory” cytokines and “human” MSCs were identified. Herein, we review the cell biology and mechanisms of chondrocyte phenotype acquisition from human MSCs in an inflammatory milieu and discuss the clinical potential of MSCs for cartilage repair.

## 1. Introduction

Defects in the cartilage are irreversible and currently difficult to repair in patients with cartilage degenerative diseases, including rheumatoid arthritis (RA) and osteoarthritis (OA), because of the poor regenerative ability of articular cartilage. In particular, activities of daily living (ADL) are impaired in patients with progressive cartilage destruction due to the long duration of the illness and articular hypofunction, resulting in deterioration in the quality of life (QOL). Therefore, it is desirable to develop a new articular cartilage regenerative therapy for cartilage degenerative diseases. Surgical approaches including microfracture, mosaic plasty, and autologous chondrocyte implantation (ACI) have been performed for cartilage defects in OA. Marrow-inducing reparative techniques, such as microfracture, aimed at stimulating chondroprogenitor cells localized in the underlying marrow, may affect their mechanical properties in the long term because cartilage defects in OA are repaired by the fibrous cartilage rather than the hyaline cartilage. Mosaic plasty, a treatment strategy involving the implantation of autologous or allogenic osteochondral grafts also has its limitations, such as loss of healthy cartilage, risk of immune response against the implanted graft, and infections [1]. ACI is the most widely used strategy for articular cartilage repair, implanting autologous *in vitro*-expanded chondrocytes. However, the drawbacks of this procedure are complexity, cost, and the loss of cartilage capacity as a result of dedifferentiation during *in vitro* expansion [2]. Although these methods exhibit measurable efficacies with reduced pain and production of cartilage-like tissue, satisfactory long-term therapeutic effects have not been obtained to date [3,4,5,6]. In patients with RA, application of these techniques, particularly mosaic plasty and ACI, is even more difficult due to the advanced degeneration of remaining cartilaginous tissues and the vulnerability of the entire subchondral bone [7]. Thus, a cell-based therapeutic approach for cartilage regeneration using mesenchymal stem cells (MSCs) has gained attention in the recent years [8,9,10,11].

MSCs are multipotent stem cells capable of differentiating into various cell types, including chondrocytes. MSC-like cells have been found in the cartilage [12,13,14] and are considered to be involved in cartilage homeostasis [15,16,17]. MSCs also possess anti-inflammatory and immunosuppressive activities and have been reported to show efficacy without serious adverse reactions in a clinical trial for graft-versus-host disease (GvHD) [18]. Although several clinical trials for articular cartilage regeneration using MSCs have been conducted with proven efficacy in some of them [19,20,21], further accumulation of evidence and development of effective methodologies are required [22]. The fundamental etiologies of RA and OA are considered to be different, and both conditions develop distinct degrees and patterns of articular cartilage impairment; however, the pathophysiology of inflammation in the affected joints is similar in both diseases [23]. Thus, when a regenerative therapy for articular cartilage using MSCs is sought, deep understanding of the mechanisms underlying the differentiation of MSCs into chondrocytes and the influences therein from the inflammatory milieu are likely to be important for the development of an efficient cell therapeutic strategy.

## 2. Inflammation in Cartilage Degenerative Disease

Although the etiologies of OA and RA differ from each other, both diseases exhibit inappropriate articular cartilage destruction, which is mainly due to elevated levels of proteolytic enzymes. RA is an autoimmune disease in which excessive inflammatory reactions resulting from abnormal immune function destroy the joints. Because biological agents targeting proinflammatory cytokines, such as TNF-α and IL-6, exhibit high efficacy in clinical practice, it is clear that inflammation has an important role in the pathological process of the disease. Synovial hyperplasia contributes to the local production of inflammatory cytokines and proteolytic enzymes that degrade the cartilage matrix. The disease characteristically involves the small joints of the hands and feet, although inflammation of larger joints is also frequent. On the other hand, although the causes of OA remain largely unknown, changes due to aging, genetic factors, excessive mechanical stresses, direct injuries, and articular surface deformation caused by trauma are considered to be involved in OA pathogenesis. In addition, the occurrence of episodic intra-articular inflammation with synovitis indicates that the synovium may also be a source of inflammatory cytokines and proteolytic enzymes because >90% of patients with OA develop synovitis [24] with infiltration of activated B and T cells [25]. Thus, as in RA, inflammation appears to be a major factor contributing to cartilage destruction and progression of symptoms in OA [26].

The proinflammatory cytokines released during synovitis, such as IL-1β and TNF-α act on chondrocytes and inhibit the production of type II collagen and aggrecan, the major components of the cartilage matrix [27,28]. In addition, they increase the release of matrix metalloproteinases and aggrecanase, enzymes that degrade the matrices, resulting in cartilage destruction [29,30,31] and disturbing the metabolic balance of the cartilage matrix [32]. The sources of these inflammatory cytokines and proteolytic enzymes are not just the synovial cells but also the chondrocytes themselves, which contribute to cartilage destruction [33]. Moreover, IL-1β and TNF-α induce apoptosis of chondrocytes by promoting the production of other proinflammatory factors such as nitric oxide (NO) and prostaglandin E2 (PGE2) [34]. Several reports have suggested that other proinflammatory factors such as IL-17, produced by T cells, have similar effects on chondrocytes [35,36] and synergize with other inflammatory cytokines (IL-1β, TNF-α, IL-6, IL-17, and oncostatin M), disturbing the anabolic/catabolic balance of the cartilages [37,38,39,40].

## 3. Molecular Mechanisms of Chondrogenic Differentiation

MSCs are multipotent stem cells capable of differentiating into chondrocytes [41]. This differentiation is an important process during cartilaginous tissue and bone formation by endochondral ossification and occurs during the development and growth of the skeletal system [42]. Majority of the molecular mechanisms are common with the chondrogenesis of adult MSCs. MSCs are present in various tissues in the adult body. Among the articular tissues, bone marrow [43], synovial membrane [44], tendon [45], meniscus [46], adipose tissue [47], and even the articular cartilage itself [15,16,17] contain MSCs. It is likely that the process of chondrogenic differentiation of MSCs is not only limited to development and growth but also involved in the repair of injured skeletal tissues such as fractured endochondral bone and the maintenance of cartilage homeostasis in adult life [12,13,14].

The process of chondrogenesis is triggered by factors such as bone morphogenetic proteins (BMPs) [48], transforming growth factor β (TGF-β) [49] and wingless-type MMTV integration site family members (Wnts) [50], leading to the expression of the master transcription factor SRY-box 9 (Sox9), which is essential for chondrocyte differentiation [51]. Sox9 controls the transcription of genes characteristic to the cartilage matrix, such as type II collagen and aggrecan, and it also suppresses the subsequent formation of hypertrophic chondrocytes [52,53]. Therefore, conditional Sox9-deficient mice are born without limbs due to the lack of cartilage matrix production [53,54]. In addition, *Sox9* has been identified as a causative gene of campomelic dysplasia, a lethal congenital bone disease accompanied by severe limb shortening in humans [55]. Sox9 induces the expression of coupling factors, l-Sox5, and Sox6, forming a transcription complex, which activates the transcription of cartilage-related genes such as *Col2a1* [53,56]. In addition, cofactors such as p300/adenosine 3',5'-cyclic monophosphate (cAMP) response-element binding protein binding protein (p300/CBP), Scleraxis/E47, Tip60, and c-Maf are also involved in transcriptional regulation [57,58,59]. On the other hand, some reports have pointed out poor correlations between the Sox9 expression levels and its target genes [60,61]. Similar to other transcription factors, phosphorylation of Sox9 has been intensively studied, and it has been observed that phosphorylation of Sox9 by protein kinase A (PKA) [62,63], cytidine 3',5'-cyclic monophosphate (cGMP)-dependent protein kinase II (cGKII) [64], and Rho kinase (ROCK) [65], increase DNA binding to target genes and the transcriptional activity of the *Sox9* gene. The regulation of Sox9 expression itself remains largely unknown. However, novel regulatory factors important for the initial differentiation of adult MSCs into chondrocytes have recently been identified, and their involvement in the regulation of Sox9 expression has been revealed. The transcription factor runt-related gene 1 (Runx1) induces Sox trio expression, *i.e.*, Sox9, Sox5, and Sox6, and directly increases the promoter activity of *Col2a1* [66]. In addition, post-transcriptional regulation mediated by microRNAs (miRNAs), small RNAs that do not encode a protein, control chondrogenic differentiation [67]. For instance, miR-145 directly targets *Sox9* [68], whereas miR-140 shows expression variation parallel to those of *Sox9* and *Col2a1* and is induced by the abovementioned Sox trio [69,70,71] contributing to both development and maintenance of cartilage [72]. Prechondrocytes differentiate into mature chondrocytes producing abundant aggrecan and collagen fibers (types II, IX, and XI). Chondrocytes begin to express alkaline phosphatase in conjunction with the transcription factors, Indian hedgehog (Ihh), and parathyroid hormone-related protein receptor (PTHrP-R) and produce type X collagen instead of type II collagen [73]. Transcription factors Runx2 and Osterix also have important roles in cartilaginous hypertrophy [74,75] and cause calcification of the matrix by promoting calcium deposition [76]. The transcription factor Runx2 promotes the differentiation of MSCs into hypertrophic chondrocytes, whereas Sox9 expression is lost during this process.

## 4. Effect of Inflammation on Chondrogenic Differentiation

Proinflammatory cytokines induce cartilage destruction by disturbing its metabolic balance through the suppression of cartilage matrix production, enhancement of production of cartilage matrix-degrading enzymes by chondrocytes, and induction of chondrocyte apoptosis. Although a number of studies have focused on the effect of inflammation on chondrocytes and the anabolic/catabolic balance of the cartilage matrix, very few reports on chondrogenic differentiation have been published so far. A PubMed search using the following medical subject headings (MeSH) terms: mesenchymal stromal cells, chondrogenesis, and cytokines, was conducted in October 2014, resulting in a total of 204 articles. The titles and abstracts were screened and nine articles relevant to “inflammatory” cytokines and “human” MSCs were identified (Table 1). Several major studies have described the molecular mechanisms behind the influence of inflammation on the MSC differentiation into chondrocytes. IL-1α, IL-1β, and TNF-α inhibit chondrogenic differentiation, based on an investigation using human MSCs [77,78,79,80,81]. Moreover, Wehling *et al.* have reported that the role of IL-1β in suppressing chondrogenic differentiation was dependent on the activation of NF-κB, given that it was cancelled by inhibition of nuclear transport of NF-κB. Similarly, the role of TNF-α in suppressing chondrogenic differentiation is also NF-κB-dependent and is caused by post-transcriptional down-regulation of Sox9 [82]. Both cytokines suppress Sox9 expression by suppressing TGF-β receptor II expression, an important initiation factor of chondrogenic differentiation, thereby suppressing the activation of Smad2/3, a downstream signaling molecule of TGF-β receptor II, and enhancing the inhibitory molecule Smad7 expression [83,84,85]. In other words, IL-1β and TNF-α inhibit chondrogenic differentiation by the inhibition of TGF-β/Smad signaling, which is an important trigger of initial chondrogenic differentiation. In addition to these findings, it has been reported that CXC chemokine ligand 7 (CXCL7), but not IFN-γ, inhibits chondrogenic differentiation [79,86].

The over-expression of IL-1 receptor antagonist (IL-1Ra) improved the ability to repair full-thickness cartilage defects formed by microfracture in animals *in vivo* [87]. We recently reported that IL-17, a key cytokine of chronic inflammation, suppressed the MSC differentiation into chondrocytes [88]. IL-17, unlike IL-1β and TNF-α, acts by the suppression of the phosphorylation of Sox9, important for transcriptional activation, with no influence on TFG-β/Smad signaling or Sox9 protein expression. The conditioned medium of synovial cells from patients with OA and synovial fluid from patients with RA were reported to strongly suppress MSC differentiation into chondrocytes [80,89,90]. Unknown factors are likely to be involved in this activity, given that inhibition of IL-1 or TNF-α leads to partial recovery of cartilage differentiation induction, but not full recovery [80]. These findings suggest that chondrocyte differentiation is suppressed in the joints of patients with OA or RA when compared with healthy joints.

**Table 1:** Effect of cytokines on chondrogenic differentiation of human mesenchymal stem cells.

Inflammatory Cytokine	Cell Source	Culture Method	Outcome	References
IL-1α	BM-MSCs	pellet culture	inhibit	[79,80]
IL-1α	BM-MSCs	PCL scaffolds	inhibit	[81]
IL-1β	BM-MSCs	pellet culture	inhibit	[77,78]
IL-17	BM-MSCs	pellet culture	inhibit	[88]
TNF-α	BM-MSCs	pellet culture	inhibit	[77,79,80]
IFN-γ	BM-MSCs	pellet culture	no effect	[79]
CXCL7	BM-MSCs	micro-mass culture	inhibit	[86]
Conditioned mediun from OA synovium	BM-MSCs	pellet culture	inhibit	[80]
RA synovial fluid	Prechondrogenic mesenchymal cells	High density culture	inhibit	[89,90]

BM-MSCs = Bone marrow derived MSCs; PCL = poly (ɛ-caprolactone).

**Figure 1:**
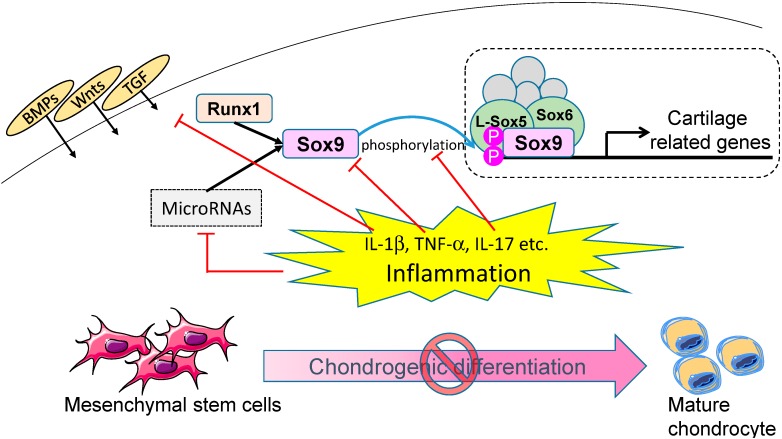
Schematic diagram of the potential role of inflammation on chondrogenic differentiation. Inflammatory mediators, such as IL-1β, TNF-α, and IL-17, contribute to the inhibition of chondrogenic differentiation of mesenchymal stem cells (MSCs) through several mechanisms. Abbreviation: BMPs, bone morphogenetic proteins; Wnts, wingless-type MMTV integration site family members; TGF, transforming growth factor; Runx, runt-related gene; Sox, SRY-box.

Given that miRNA (miR-140, -145 and -199a) expression levels, which have been reported to be involved in chondrogenic differentiation, are altered by IL-1β stimulation, changes in the miRNA network are also likely to be a mechanism by which inflammation suppresses chondrogenic differentiation [70,91,92]. Thus, MSC differentiation into chondrocytes is strongly suppressed in an inflammatory milieu by the suppression of both Sox 9 expression and transcriptional activation, mediated by proinflammatory cytokines including IL-1, TNF-α, and IL-17 (Figure 1).

## 5. Clinical Application of Mesenchymal Stem Cells (MSCs)

Clinical trials of cell therapies using MSCs for articular cartilage regeneration in patients with OA have been conducted and have been proven successful in some cases [93]. In particular, the result from a proof-of-concept clinical trial reported by Jo *et al.* is noticeable; intra-articular injection of adipose-derived MSCs was demonstrated to lead to apparent repair by the hyaline cartilage-like cartilaginous tissue, based on arthroscopic and histopathological evaluations [19]. The results obtained from these clinical trials have raised hopes for the potential of MSCs in cartilage regenerative therapies. However, additional studies involving various protocols are still ongoing (National Library of Medicine ClinicalTrials.gov, available online: http://clinicaltrials.gov/) due to insufficient evidence and unsatisfactory regenerative effects. Development of safer and more efficient therapeutic protocols for the establishment of cartilage regenerative therapies using MSCs still remains a challenge [5,6].

Studies focused on improving the efficiency of MSC differentiation into chondrocytes are also being aggressively conducted to develop more effective cartilage regenerative therapies using MSCs. Various additional factors including TGF-β1–3; BMP-2, -4, -6, -7; fibroblast growth factor-2 (FGF-2); insulin-like growth factor-1 (IGF-1); insulin; and PTHrP promote MSC differentiation into chondrocytes [94]. Furthermore, dexamethasone, adenosine 5'-triphosphate (ATP), stromal-derived factor-1β (SDF-1β), growth and differentiation factor-5 (GDF-5), FGF-18, among others, have also been reported to positively regulate MSC differentiation into chondrocytes [9]. TD-198946 [66] and kartogenin (KGN) [95] were recently reported as synthetic low molecular weight compounds with chondrogenic differentiation promoting activity. These compounds were confirmed to possess cartilage regeneration effects in animal OA models by acting on the transcription factor Runx1 involved in initial cartilage differentiation. In addition, curcumin was found to rescue the inhibitory effect of IL-1β on chondrogenic differentiation of human MSCs *in vitro* [96]. Chondrogenic differentiation is also enhanced by environmental stimulation such as hypoxic conditions [97,98] and mechanical stress [99]. Moreover, hypoxia has been shown to reduce the inhibitory effect of IL-1β on chondrogenesis *in vitro* [78].

MSCs also possess immunosuppressive and anti-inflammatory properties depending on their trophic functions, secreting a number of soluble mediators such as IL-10, TGF-β and indoleamine 2,3-dioxygenase (IDO) [100,101]. Injection of MSCs prevented the irreversible damage of bone and cartilage in type II collagen-induced arthritis (CIA) based on their immuno-modulatory function and ability to induce regulatory T cells [102]. In humans, the use of MSCs has been reported to be safe and efficacious in a variety of autoimmune diseases such as GvHD [103], multiple sclerosis [104], and systemic erythematosus [105]. Therefore, intra-articular injection of MSCs is expected to be beneficial for immune regulation and cartilage regeneration. However, relatively large numbers of cells are required to express the anti-inflammatory effect *in vivo* and their retentivity and undifferentiated status, after intra-articular injection, is poorly known.

**Figure 2:**
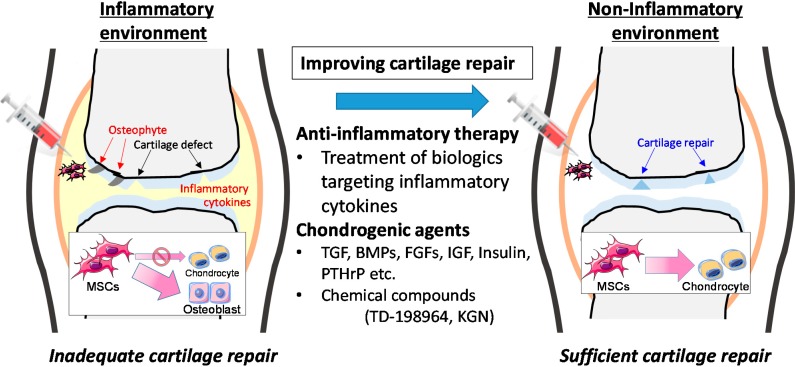
Strategies for efficient cartilage repair therapy by intra-articular injection of mesenchymal stem cells (MSCs). The inflammatory environment is present in the joints affected by rheumatoid arthritis (RA) or osteoarthritis (OA). Chondrogenic differentiation of intra-articular injected MSCs is inefficient under inflammatory conditions. Therefore, preoperative treatment with anti-inflammatory drugs and/or chondrogenic agents should be important for efficient cartilage repair therapy.

In several clinical trials, MSCs injected into the joints by the least-invasive intra-articular method, were expected to differentiate into chondrocytes locally in the joint. Whether or not the intra-articular environment of the joints is suitable for chondrogenic differentiation of MSCs is a major concern. As mentioned earlier, cartilage regenerative therapy is likely to be indicated when the affected joints in patients with OA and RA present with inflammatory reactions. However, the inflammatory milieu, including proinflammatory cytokines, strongly inhibits MSC differentiation into chondrocytes. On the other hand, we had previously demonstrated the influence of inflammation (proinflammatory cytokines) on MSC differentiation into osteoblasts, resulting in the formation of calcifications [106,107]. These findings indicate the risk of developing osteophytes and ectopic calcifications when MSCs are injected into the joint in an inflammatory milieu. Therefore, sufficient suppression of articular inflammation prior to the operation is important for efficient cartilage regeneration while using MSCs in cartilage regenerative therapy. This can be achieved by a combination of an existing anti-inflammatory agent or a biologic targeting proinflammatory cytokine and a chondro-enhancing agent (Figure 2). Further evidence and studies are warranted for the improvement and maintenance of intra-articular environment suitable for cartilage regeneration.

## 6. Conclusions

The inflammatory milieu is known to cause cartilage destruction by causing disturbances in the anabolic/catabolic balance of the cartilage matrix. The inflammatory milieu has also been understood to exert a suppressive action during the process of differentiation from progenitor MSCs to chondrocytes. Therefore, sufficient suppression of articular inflammation prior to the operation may be important for efficient regeneration of the cartilage in regenerative therapies using MSCs. While continuous studies to develop more efficient techniques for chondrogenic differentiation using MSC-based cell therapies are important, it will also be necessary to establish a protocol including the use of existing agents and development of novel agents for the improvement and maintenance of the intra-articular environment, where MSCs are transferred, to be suitable for cartilage regeneration.
